# Augmentation of Antipsychotic Medications with Low-Dose Clozapine in Treatment-Resistant Schizophrenia—Case Reports and Discussion

**DOI:** 10.1155/2021/5525398

**Published:** 2021-06-19

**Authors:** Zoe Harrison, Owen Haeney, William Brereton

**Affiliations:** ^1^University of Adelaide, Faculty of Health and Medical Sciences, 4 North Terrace, Adelaide South Australia 5000, Australia; ^2^Forensic Mental Health Service/Northern Adelaide Local Health Network, James Nash House, 140 Hilltop Drive, Oakden, Adelaide South Australia 5086, Australia; ^3^University of New South Wales, Wallace Wurth Building, 18 High St., Kensington, Sydney NSW 2052, Australia

## Abstract

Treatment resistance in schizophrenia is often encountered in clinical practice, with clozapine usually recommended as the appropriate therapy. However, where clozapine proves ineffective or cannot be tolerated due to side effects, treatment options are limited. In patients within forensic mental health services, residual symptomatology often presents a barrier to discharge and can have lasting effects on prospects for rehabilitation as well as risk to self and others. This paper presents a review of the relevant literature and three cases of a novel approach, utilising clozapine in doses usually considered subtherapeutic, in combination with the primary antipsychotic treatment. In all three patients, it improved clinical efficacy as well as tolerability, resulting in improvement that allowed discharge from the forensic hospital.

## 1. Introduction

Schizophrenia is a common mental disorder with lifetime prevalence of 1% of the population [1]. Treatment adopts a biopsychosocial approach. The mainstay of pharmacotherapy is antipsychotic medication, which can be effective in ameliorating symptoms. However, up to 30% of patients meet the criteria for treatment resistance [2–4], conventionally defined as failure to respond to treatment with two different antipsychotic medications despite adequate doses for a sufficient trial period, usually of at least 4-6 weeks [1, 5]. Where the diagnosis is confirmed, and no comorbidity is present, most guidelines recommend immediate treatment with clozapine. The duration of untreated psychosis is negatively correlated with outcome [6–9], and there is little evidence for efficacy of further trials of conventional antipsychotic medications or polypharmacy.

Clozapine is specifically licensed for treatment-resistant schizophrenia, with meta-analyses consistently demonstrating that its efficacy is superior to other antipsychotic medications [10–13]. However, clozapine itself is associated with significant risk of side effects at usual therapeutic doses. Its prescription requires strict monitoring through an authorized monitoring service due to risks of agranulocytosis. Additional potential side effects include weight gain, myocarditis, cardiomyopathy, constipation, hypersalivation, reduced seizure threshold, or sedation. As a result, patients may be reluctant to accept it despite its efficacy [14]. Up to 45% of people who commence clozapine discontinue it within two years, with over half doing so due to intolerable side effects [15].

Even where clozapine is tolerated at therapeutic doses, only 40% of treatment-resistant patients respond adequately [16]. Overall, between 12 and 20% of people with schizophrenia may therefore be classed as ultraresistant [17]. In that eventuality, it is common practice to augment clozapine with other treatments, including other antipsychotic medications, mood stabilising drugs, anticonvulsants, ECT (electroconvulsive therapy), or novel alternatives, often with the aim of reducing clozapine dose to enhance tolerability [18–23]. It would be beneficial to construct an algorithm for stepwise management of those who are treatment resistant, and one online survey provides some reassurance of standardised management approaches amongst clinicians [24]. However, the evidence supporting clinical response from these methods is insufficient to develop a definite hierarchy for further treatment, and, in clinical practice, results are often underwhelming. Where clozapine is declined or cannot be tolerated, treatment options are even more limited. As a result, patients are often left with significant residual disability.

Forensic mental health services work at the interface of mental health and the criminal justice system. Although systems and legislation can vary across jurisdictions, those who commit serious offences driven by mental illness are usually subjected to compulsory detention in a secure hospital. There, they can access treatment for their mental illness and any associated comorbidity. Frequently, in order to achieve discharge from secure care, those patients will have to convince a judicial body, such as a court or tribunal, that they are safe to be released. Significant residual symptoms can therefore be a substantial barrier to discharge.

While augmenting clozapine with other therapeutic options has been the mainstay of management in treatment resistant schizophrenia, one novel approach that has been suggested is using low-dose clozapine to augment the primary antipsychotic treatment [25]. The rationale for this treatment is that there may be benefit even at low doses of clozapine, which may be better tolerated, particularly where it complements the receptor profile affected by the primary antipsychotic drug. In clinical practice, one of the authors has used this approach on three occasions, each time with significant success.

## 2. Case Presentations

### 2.1. Case One

A single, unemployed male in his early 40s was diagnosed with paranoid schizophrenia. He had been found by the court to be not guilty by reason of mental impairment, being unwell at the time of his latest offence, experiencing bizarre persecutory delusions and command hallucinations of a homicidal and suicidal nature. He had a long history of violent offences and noncompliance with medication. His illness was exacerbated by drug abuse, particularly amphetamines.

Clozapine was initiated when his schizophrenia was deemed treatment resistant. It was slowly titrated to a total dose of 400 mg per day, at which his serum levels ranged between 510 and 975 micrograms per litre. He responded, but later complained of restless legs, which contributed to episodes of nonadherence to medication. Clozapine was reduced to 350 mg per day, but similar problems continued, and it was gradually withdrawn, leading to deterioration in his mental state. Paliperidone long-acting injection was initiated instead and maintained at 150 mg monthly. Although his mental state improved, it fluctuated greatly, making discharge unsafe. As an adjunct to his primary treatment with paliperidone, clozapine was reintroduced and titrated to 150 mg daily, later 200 mg after slight deterioration in mental state. At this dose, his serum levels ranged between 137 and 254 micrograms per litre, substantially below the levels usually regarded as therapeutic.

There was a significant improvement in his mental state and insight. He did continue to experience mild persecutory ideas and auditory hallucinations, but they did not fluctuate as they had before. He went on to use amphetamines twice during his rehabilitation; however, they did not have the severe negative impact on his mental state as previously. His insight allowed him to admit to his use and continues to work with services on relapse prevention. He was subsequently discharged.

### 2.2. Case Two

The second case was a male patient in his late 40s, diagnosed with paranoid schizophrenia. He suffered from bizarre and grandiose delusions and prominent auditory hallucinations. These had been poorly responsive to treatment, exacerbated by poor medication adherence and avoidance of community follow-up. He had a significant history of violence and was found not guilty by reason of mental impairment following a charge of murder.

During his admission to the forensic hospital, his mental state fluctuated greatly. Despite periods of stability, stress caused a rapid return to significant psychosis, often associated with agitation, verbal aggression, and threats.

Multiple antipsychotics and combinations were tried without sustained success. Clozapine was commenced and slowly titrated according to the usual introductory regime. Unfortunately, even within the first month, the patient complained of troublesome sedation which he was unwilling to tolerate, despite reassurance that this would improve. Even at doses as low as 100 mg per day, with serum levels peaking at 107 micrograms per litre, he was unwilling to continue. Treatment with risperidone long-acting injection up to 100 mg, olanzapine 10 mg daily, and allopurinol 300 mg twice daily maintained some stability but could not prevent further acute relapses. Two years after the first trial of clozapine, an attempt to reintroduce it as monotherapy was made but the patient refused to continue, due to similar side effects, within several days. However, he agreed to take clozapine as an adjunct at a lower dose of 50 mg daily. His serum levels lay in the region of 50–61 micrograms per litre at this dose.

Following this sustained addition of clozapine, his mental state became more resilient. He experienced episodes of considerable stress without decompensating as he would have previously. This allowed him to start a slow program of leave out of the forensic hospital and progress through community-based rehabilitation and eventual discharge.

### 2.3. Case Three

The third case was a male in his late 40s with treatment-resistant paranoid schizophrenia, admitted to forensic services following a violent offence. A history of amphetamine abuse and nonconcordance with oral antipsychotic medication in the community exacerbated his relapsing, remitting psychotic symptoms.

Upon starting clozapine, he improved clinically and began to engage in rehabilitation. His mental state stabilised on a dose of 300 mg per day (with serum levels 337–632 micrograms per litre). He was discharged to a step-down community forensic rehabilitation unit. While he was there, he did not experience psychotic symptoms, but his insight remained poor, and he made it clear he would not be compliant with clozapine if not directly monitored by staff. He objected to the side effects, particularly weight gain, sedation, and hypersalivation. As a result, he was weaned off clozapine and commenced on aripiprazole long-acting injection 400 mg every four weeks.

He did not suffer any acute relapse of symptoms. However, disorganisation and negative symptoms gradually reemerged, including amotivation, poor concentration, and low mood. His functioning deteriorated. He agreed to start a low dose of adjunctive clozapine at 50 mg daily (correlating with serum levels between 57 and 121 micrograms per litre). Objectively, this resulted in considerable improvement and allowed progress towards discharge. Subjectively, he was not convinced of improvement, but he was prepared to tolerate it, as he was no longer troubled by side effects.

## 3. Discussion

A literature review using databases including Cochrane, Medline, Embase, and PsychInfo identified limited case reports suggesting benefit from utilizing low-dose clozapine to augment the primary antipsychotic treatment, which was mainly aripiprazole. Stoner and colleagues discussed a case of treatment-resistant schizophrenia whereby reduction of clozapine dose to subtherapeutic levels removed unwanted sedation, while maintaining its effectiveness [26]. This patient was previously deemed incompetent to undergo legal proceedings for a crime related to assaultative behaviour, subsequently leading to his psychiatric hospital admission. The authors commented that as clozapine usually required slow-dose titration, accepting subtherapeutic levels of clozapine meant rapid management could be instigated. This was particularly advantageous as the duration of untreated illness is correlated with poorer outcomes [9] and, in patients with a forensic history, may pose an immediate risk to patient and staff safety.

Sepede and colleagues reported a case where traditionally dosed clozapine was the only medication noted to ever alleviate symptoms in a patient with treatment-resistant schizophrenia but also caused excessive sedation and an episode of myoclonus. Due to patient preference for convenience and reliability in drug administration, a combination regimen with long-acting injectable (LAI) aripiprazole facilitated the successful use of subtherapeutic doses of clozapine [27]. The authors questioned whether the use of low-dose clozapine could potentially allow relaxation of the strict monitoring regime and thus could encourage patients to be more accepting of a clozapine trial. The use of long-acting injectable antipsychotic drugs with addition of low-dose clozapine would be particularly promising and may provide an alternative option to manage issues of adherence commonly seen in antipsychotic prescribing.

In the case series by Lim and colleagues, the potential benefit for a low-dose clozapine regimen is also conveyed in affective psychiatric disease featuring psychosis [28]. The authors questioned whether included patients may have improved taking clozapine monotherapy, as would have been the next standard therapeutic step after multiagent failure. However, when used to augment another agent, subtherapeutic doses of clozapine were given in two of the three cases, with positive outcomes. The authors postulated that the low doses used were a protective factor against dose dependent adverse effects, including sedation, constipation, and seizures.

When added to other antipsychotic treatment, low-dose clozapine may also assist in reducing dose-related side effects of the non-clozapine antipsychotic agent. Hung and colleagues presented a case of schizophrenia where aripiprazole therapy provided vast symptomatic improvement but induced Parkinsonism and severe akathisia. Clozapine monotherapy was initiated at subtherapeutic levels with low-dose aripiprazole added again due to past efficacy, with no return of extrapyramidal side effects [29].

The literature review and case reports summarise the evidence and rationale for a novel approach to treating the so-called ultraresistant schizophrenia, particularly when clozapine cannot be tolerated in the doses required to establish therapeutic serum levels. Use of low-dose clozapine in combination with an alternative primary antipsychotic drug may offer an effective compromise of efficacy and tolerability. It is important to note that usual monitoring blood tests, such as white cells and neutrophils, are still required.

Clozapine binds weakly to D1 and D2 receptors, with affinity for D4, 5-HT2, 5-HT3, alpha-1, alpha-2, H1, and M1 receptors. Metabolic risks are believed to be due to the strong blockade of 5HT2C and H1 receptors [30], and stimulation of the enzyme AMPK (adenosine monophosphate-activated protein kinase) in the hypothalamus, reversing the effects of leptin [31]. Clozapine's rapid dissociation from D2 receptors is credited for its lower risk of extrapyramidal side effects [32]. Its wide spectrum of action is thought to give benefit via various neurotransmitter systems with potential advantage when used alongside other “atypical” antipsychotic medications such as risperidone, quetiapine, and olanzapine.

Newer agents including amisulpride (a highly selective D2 and D3 receptor blocker), and aripiprazole (a partial agonist of both dopaminergic and serotonergic receptors, assisting in hyper- and hypodopaminergic dysfunction in schizophrenia), may have a synergistic effect with the receptor profile of clozapine. Indeed, the effects of aripiprazole on 5HT2C and 5HT1A receptors are posited to protect against clozapine-induced weight gain, dyslipidaemia, and hyperglycaemia associated with risk of developing diabetes [33–36].

Despite the lowered risk of side effects owing to subtherapeutic dosing of clozapine, this strategy may not be without undesirable effects. There have been a handful of cases reported where low-dose clozapine monotherapy has resulted in adverse effects despite subtherapeutic levels, including seizures [37], persistent tachycardia [38], and tardive dystonia [39]. Additionally, consideration must be given to drug-to-drug interactions when using multiple agents. Ray and Munshi presented a case where adding low-dose clozapine to baseline amisulpride treatment aggravated existing akathisia, with new onset dystonia and hypersalivation [40]. The authors postulated that these adverse effects were due to increased serum amisulpride levels due to interaction with clozapine, which has been reported previously [41]. The removal of amisulpride and change to clozapine monotherapy resulted in complete remission of adverse effects.

The current literature highlights the difficulty related to treatment in those with treatment-resistant schizophrenia where guidelines are less defined, stressing the need for further research into personalised management to the individual. Further research comprising more robust methodologies, longer follow-up periods, and larger and more diverse cohorts is necessary.

These case reports of 3 patients with treatment-resistant schizophrenia demonstrate the potential feasibility and efficacy of augmenting standard antipsychotic monotherapy with low-dose clozapine. This adds to the growing body of literature, with few reported cases exploring this therapeutic method previously.

## Data Availability

The patient notes used to support the findings of this study are restricted by the Central Adelaide Local Health Network HREC in order to protect patient privacy.
